# National Antimicrobial Consumption: Analysis of Central Warehouses Supplies to In-Patient Care Health Facilities from 2017 to 2019 in Uganda

**DOI:** 10.3390/tropicalmed6020083

**Published:** 2021-05-19

**Authors:** Juliet Sanyu Namugambe, Alexandre Delamou, Francis Moses, Engy Ali, Veerle Hermans, Kudakwashe Takarinda, Pruthu Thekkur, Stella Maris Nanyonga, Zikan Koroma, Joseph Ngobi Mwoga, Harriet Akello, Monica Imi, Freddy Eric Kitutu

**Affiliations:** 1 Department of Pharmacy, Mbarara University of Science and Technology, Mbarara, P.O. Box 1410 Mbarara, Uganda; 2 Africa Centre of Excellence for Prevention and Control of Transmissible Diseases (CEA-PCMT), University Gamal Abdel Nasser, Conakry, PB: 4099 Maferinyah, Guinea; adelamou@gmail.com; 3 Centre National de Formation et de Recherche en Santé Rurale (CNFRSR) de Maferinyah, PB: 4099 Maferinyah, Guinea; 4 Ministry of Health and Sanitation, 00232 Freetown, Sierra Leone; franqoline@gmail.com (F.M.); zikankoroma@gmail.com (Z.K.); 5 College of Medicine & Allied Health Sciences, University of Sierra Leone, 00232 Freetown, Sierra Leone; 6 Médecins Sans Frontières–Operational Centre Brussels, Medical Department, Operational Research Unit (LuxOR), Luxembourg De Manstraat 6, 2100 Deurne, Brussels, Belgium; engy.ali@luxembourg.msf.org (E.A.); Veerle.HERMANS@luxembourg.msf.org (V.H.); 7 Centre for Operational Research, International Union Against Tuberculosis and Lung Disease, 75006 Paris, France; ktakarinda@theunion.org (K.T.); Pruthu.tk@theunion.org (P.T.); 8 Clinical Epidemiology Unit, College of Health Sciences, Makerere University, P.O. Box 7072 Kampala, Uganda; snanyonga2012@gmail.com; 9 World Health Organisation County Office, P.O. Box 24578 Kampala, Uganda; mwogaj@who.int; 10 Ministry of Health Uganda, P.O. Box 7272 Kampala, Uganda; harakello@gmail.com; 11 Enabel, The Belgian Development Agency, P.O. Box 40131 Kampala, Uganda; monicaimialt@gmail.com; 12 Sustainable Pharmaceutical Systems (SPS) Unit, Pharmacy Department, School of Health Sciences, Makerere University, P.O. Box 7072, Kampala, Uganda; kitutufred@gmail.com

**Keywords:** antimicrobial resistance, antimicrobial consumption, antimicrobial stewardship, Uganda, health facilities, antimicrobials, defined daily doses, operational research, SORT-IT

## Abstract

Antimicrobial consumption (AMC) surveillance at global and national levels is necessary to inform relevant interventions and policies. This study analyzed central warehouse antimicrobial supplies to health facilities providing inpatient care in Uganda. We collected data on antimicrobials supplied by National Medical Stores (NMS) and Joint Medical Stores (JMS) to 442 health facilities from 2017 to 2019. Data were analyzed using the World Health Organization methodology for AMC surveillance. Total quantity of antimicrobials in defined daily dose (DDD) were determined, classified into Access, Watch, Reserve (AWaRe) and AMC density was calculated. There was an increase in total DDDs distributed by NMS in 2019 by 4,166,572 DDD. In 2019, Amoxicillin (27%), Cotrimoxazole (20%), and Metronidazole (12%) were the most supplied antimicrobials by NMS while Doxycycline (10%), Amoxicillin (19%), and Metronidazole (10%) were the most supplied by JMS. The majority of antimicrobials supplied by NMS (81%) and JMS (66%) were from the Access category. Increasing antimicrobial consumption density (DDD per 100 patient days) was observed from national referral to lower-level health facilities. Except for NMS in 2019, total antimicrobials supplied by NMS and JMS remained the same from 2017 to 2019. This serves as a baseline for future assessments and monitoring of stewardship interventions.

## 1. Introduction

Antimicrobial resistance (AMR) is a major global health threat with predicted impact of social, economic, and environmental dimensions [1]. Common infections have become difficult to treat due to resistance to available antimicrobial agents, resulting in high morbidity and mortality rates [1]. Health systems in low and middle-income countries (LMIC) bear a bigger brunt due to the increased cost of treating resistant infections, longer hospital admissions, need for additional laboratory tests, and expensive antibiotics. Moreover, LMICs are more vulnerable to the AMR burden because of the inadequate infrastructure and severe shortage of human and financial resources for health systems [2,3]. For instance, of the predicted 10 million annual deaths attributable to AMR by 2050, 80% is expected to occur in LMICs in Africa and Asia, and their gross domestic product (GDP) and livestock output could contract by up to 5% and 7.5%, respectively, by 2050 [4]. 

AMR is rife in Uganda as shown by the 2015 situational analysis where high resistance to commonly used antibiotics, including cotrimoxazole, ampicillin, and ceftriaxone, was documented [5]. Additionally, high prevalence of multi-drug resistant bacteria such as methicillin-resistant *Staphylococcus Aureus* and extended-spectrum beta-lactamase (ESBL)-producers was reported [5,6]. To address the AMR problem globally, the World Health Organization (WHO) global action plan (GAP) on AMR proposed surveillance and optimization of antimicrobial use as one of five pillars for adoption and implementation [7]. In line with the WHO GAP, Uganda launched a national action plan (NAP) to slow down the progression of AMR in 2018. Surveillance of AMR and optimization of antimicrobial use in human, animal and environmental health are key priority interventions in Uganda’s AMR NAP [8].

Antimicrobial consumption (AMC) as a driver for the emergence and spread of AMR is a well-established connection [9,10]. Efforts to optimize AMC and use also referred to as antimicrobial stewardship (AMS), are expected to contribute to reduced emergence and spread of resistant microbes. However, as a prerequisite, it is important to quantify and understand the extent of antimicrobial exposure in human, animal, and environmental health. In 2016, WHO published a methodology for AMC surveillance at national, regional and global level based on quantification of defined daily dose (DDD) and standardizing the estimates per population or hospital activity. This method categorizes antimicrobials by the Anatomical, Therapeutic, Chemical (ATC) coding system, which gives each antimicrobial a unique code based on its pharmacological and therapeutic use [11]. The WHO also proposed the Access, Watch and Reserve (AWaRe) classification of antimicrobials in the 2017 edition of the Essential Medicine List (EML). These provide a framework for analyzing AMC and are used to inform stewardship actions to expand availability to life saving antimicrobials where they are needed, promote their judicious use and restrict excessive use with the ultimate aim to preserve the effectiveness of last-resort antibiotics [12,13]. 

Currently, there is no systematically collated information on the quantities and nature of antimicrobials consumed at both national and sub-national levels in Uganda. Additionally, there is no standardized system for monitoring AMC and use and thus, no clear basis to evaluate ongoing AMS interventions nor to recommend and target new interventions. The health commodity supply chain in Uganda consists of both the government-owned procurement, warehousing and distribution under the National Medical Stores (NMS), the private-not-for-profit (PNFP) mechanism under the Joint Medical Stores (JMS), and other private sector pharmaceutical firms [14]. 

NMS and JMS together account for a substantial amount of antimicrobials supplied to health facilities in Uganda. Additionally, they reasonably represent the health commodity supply chain that mirrors Uganda’s mixed health system where public and privately-funded healthcare co-exist [14]. Therefore, this study analyzed the total quantity of antimicrobials supplied by NMS and JMS to inpatient health facilities from 2017 to 2019, identified the top 10 antimicrobials and determined the number of DDDs consumed by level of care of health facilities. Furthermore, the study determined the proportion of AMC by AWaRe classification, calculated the AMC density using hospital activity units and classified the antimicrobials supplied by pharmacological group. 

## 2. Materials and Methods

### 2.1. Study Design and Period

This was a descriptive cross-sectional study of aggregated data of antimicrobial products supplied by central warehouses; NMS and JMS to 442 hospitals and Level IV health centers in Uganda, from 2017 to 2019. The number of facilities from each level of care included in the study are shown in Table 1.

### 2.2. Study Setting 

#### 2.2.1. General Setting

Uganda is a low-income country in East Africa, with an estimated population of 41.6 million people [15]. It is served by a mixed health system where commercialized provision healthcare services co-exists with free or low-cost public sector [14]. Overall, 52% of all the hospitals and health facilities in the country are public, 41% are private-not-for-profit, and 7% are private-for-profit [16]. 

The health services are delivered through a tiered system, with health center, Level II as the lowest rung of the formal health system. The higher level health facilities progressively provide a broader scope of health services and serve a higher number of people through to the national referral hospital level, as shown in Table 1. The community health structure also exists and it is based on volunteer health team members. 

**Table 1:** Levels of health care delivery and number of health facilities supplied by NMS and JMS included in the study, Uganda, from 2017 to 2019.

Levels of Health Care	Type of Service	Services Provided	^1^ NMS	^2^ JMS
National Referral Hospital (NRH)	Tertiary(specialized)	Inpatient; Outpatient care	2	
Regional Referral Hospital (RRH)	Tertiary	Inpatient; Outpatient care	18	4
General Hospital (GH)	Secondary	Inpatient; Outpatient care	50	97
Health centre (HC) IV	Secondary	Inpatient; Outpatient care	179	92
Health centre III	Primary	Outpatient care only		
Health centre II	Primary	Outpatient care only		
		Total	249	193

^1^ National Medical Stores; ^2^ Joint Medical Stores.

#### 2.2.2. Specific Setting

Uganda’s health commodity supply chain system mirrors the existing mixed health system with both public and private providers. NMS and JMS are two of the pinch-point warehouses that handle supply chain functions for a substantial proportion of health commodities supplied in Uganda. For instance, for the financial year of 2019/2020, the turnover of NMS on essential medicines and health supplies (EMHS) was USD 97.5 million (UGX 363 billion) and that of JMS was USD 24.7 million (UGX 92 billion) [17,18,19]. The NMS is a government parastatal body established by an act of parliament to procure, store and distribute essential medicines, health supplies, and vaccines to all public health facilities, and JMS is a private-not-for-profit warehouse that procures and supplies health commodities to private-not-for profit health institutions, private, and government institutions [14]. 

### 2.3. Study Sites

The study included antimicrobials supplied by NMS and JMS to health facilities from the national referral hospital level down to Level IV health centers. Only health facilities that provide inpatient care were included because the current study analyzed antimicrobial exposure per health facility activity unit expressed in terms of number of patient days and admissions for standardization. Health centers, Level III and II, were excluded from the study as they only provide outpatient care services. Table 1 presents the health facilities that were included in the study.

### 2.4. Study Procedure, Data Sources, and Variables 

We adopted the WHO global antimicrobial resistance surveillance systems (GLASS) guidelines for AMC surveillance in hospitals [20], that uses the ATC/DDD methodology. The DDD is a measure used to quantify antimicrobial consumption and is assigned to each medicinal substance taking into account the route of administration. DDD stands for the average maintenance dose per day for a medicine used for its main indication in adults. The ATC system categorizes medicinal substances into different groups according to the organ or system on which they act and therapeutic, pharmacological and chemical properties [11]. 

The data collection included all antimicrobials classified by the ATC classification system under the ATC groups J01 which are antibacterials for systemic use, including oral, parenteral, and inhalation formulations and P01AB, which includes Nitroimidazole derivatives for protozoal diseases. Antibiotics used for local therapy, for example, topical creams, and eye and ear drops were excluded. Health facility activity was measured by number of patient days and number of admissions. These were obtained from the National Health Management Information System (HMIS) based on the District Health Information Software 2 (DHIS2) platform.

### 2.5. Data Collection, Sources, and Variables

The raw dataset in *Microsoft Excel (MS Excel)* software of antimicrobials supplied to study health facilities from 2017 to 2019 was obtained from the NMS and JMS. These were extracted from their internal electronic databases by the staff of the warehouses. The variables extracted included year of provision or supply, name and level of the facility, the warehouse unique product code, product name, generic name, unit of measure, route of administration, pack size, and number of packs supplied and received. 

Data were collected, cleaned, checked for completeness, and sorted by a five-man team of trained enumerators from November 2019 to June 2020. The assigned DDD per antimicrobial product and the WHO AWaRe class were then added as separate columns to the dataset. DDD per pack of each antimicrobial product was calculated. 

### 2.6. Data Analysis and Statistics

Data were sorted and re-organized using *MS Excel* (version 2010) and additional variables derived to match the variables in the WHO methodology for antimicrobial consumption in hospitals and then imported into IBM SPSS Statistics 23 for analysis. First, we calculated the total quantity of each antimicrobial product supplied by NMS and JMS per year in grams or unit doses for combined products. Second, the total amounts of each antimicrobial was converted to DDD equivalent by dividing the total amount of a product in grams consumed in a given period by the WHO-assigned DDD value, (WHO-Collaborating Center 2019 values [20]) as in the equation below.

Number of DDDs = grams of active substance/substance − specific DDD. 

We determined the total antimicrobial consumption in DDDs distributed by NMS and JMS to inpatient health centers and proportions of the top 10 antimicrobials distributed in 2017, 2018, and 2019, respectively. 

Third, we determined the total number of DDD per year supplied at each level of care of health facilities, namely, national referral hospital, regional referral hospital, general hospital and Level IV health centers. Fourth, we summarized the health facility activity units for the study period in terms of total number of patient days and number of admissions per year. We then adjusted the total number of DDDs per health facility by dividing with 100 patient days or 100 admissions to obtain AMC density. The exposure of antimicrobial use was expressed as DDD per 100 patient days and DDD per 100 admissions for each year, which generated differences in consumption between health facilities of different levels of care, as in the equation below.

$$\mathrm{AMC}\;\mathrm{density}\;=\frac{\mathrm{Number}\;\mathrm{of}\;\mathrm{DDD}\;\mathrm{of}\;\mathrm{antimicrobials}\;\mathrm{for}\;\mathrm{time}\;\mathrm{period}\;\mathrm{multiplied}\;\mathrm{by}\;100\;}{\mathrm{Quantity}\;\mathrm{of}\;\mathrm{hospital}\;\mathrm{activity}\;\mathrm{indicator}\;\mathrm{for}\;\mathrm{the}\;\mathrm{same}\;\mathrm{time}\;\mathrm{period}\;}$$

Comparison was made between health facilities’ level to assess antimicrobial consumption using the Chi-square test and 95% confidence levels, a level of significance offset at *p* ≤ 0.05. Lastly, we determined the proportions of AMC by the AWaRe and pharmacological groupings.

## 3. Results

### 3.1. Antimicrobial Products Supplied by NMS and JMS in Uganda from 2017 to 2019

There were 92 products supplied by NMS and JMS over the three years, belonging to different pharmacological classes including nitromimidazole, tetracyclines, amphenicols, penicillins, cephalosporins, carbapenems, sulphonamides and trimethoprim, macrolides and lincosamides, aminoglycosides, fluoroquinolones, glycopeptides, nitrofurans, and linezolid. The full list of products, their strengths, route of administration, dosage form, pack size, AWaRe class, and quantity in DDDs is availed in Appendix A.

### 3.2. Total Defined Daily Doses of Antimicrobials per Year from 2017 to 2019

**Figure 1:**
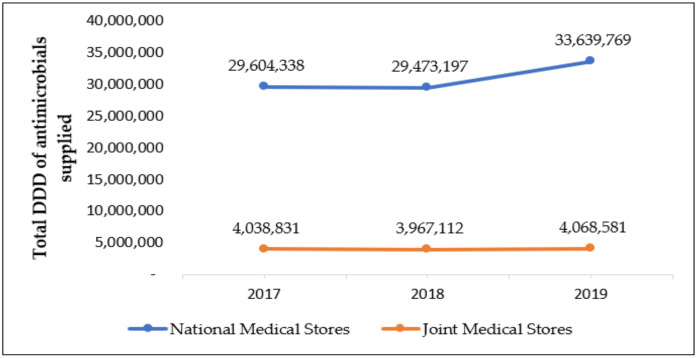
Total daily defined doses (DDD) of antimicrobials distributed from NMS and JMS from 2017 to 2019, Uganda.

The total quantity of antimicrobials in DDDs supplied by both NMS and JMS to the 442 health facilities was 33,643,169, 33,440,308, 37,708,350 in 2017, 2018, and 2019, respectively. See Figure 1 for details. NMS supplied eight times as much antimicrobials as JMS. 

The majority of NMS products were for oral route of administration (59%), followed by parenteral route (41%), and only one product for rectal route. From JMS, 86% were oral formulations, and 14% were parenteral. Ceftriaxone was the most supplied parenteral, accounting for 48% of parenteral antimicrobials from NMS and 43% from JMS. This was followed by gentamicin (14% NMS, 19% JMS) and metronidazole (13% NMS, 12% JMS).

### 3.3. Top 10 Antimicrobials Supplied by NMS and JMS to Health Facilities from 2017 to 2019

Overall, amoxicillin was the most supplied antimicrobial from NMS, accounting for 39%, 38%, and 26% in 2017, 2018, 2019, respectively, and from JMS 16%, 18%, and 19%, respectively (Table 2).

### 3.4. Analysis of Total DDD Supplied to Each Level of Care of Health Facilities per Year

**Figure 2:**
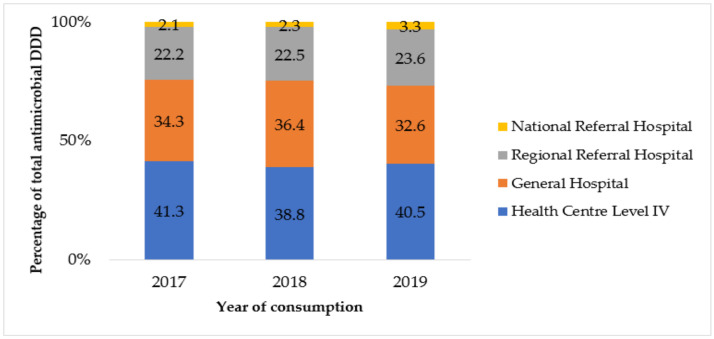
Annual total defined daily doses (DDD) supplied by National Medical Stores stratified by level of care in Uganda, 2017 to 2019.

**Figure 3:**
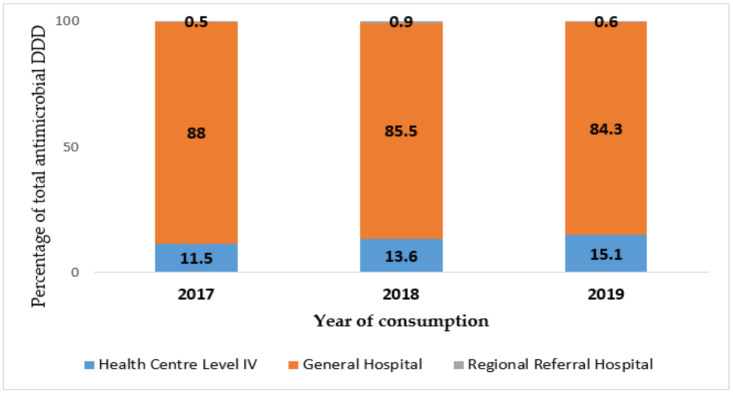
Annual total defined daily doses (DDD) supplied by Joint Medical Stores stratified by level of care in Uganda, 2017 to 2019. Level of care: blue = Level IV health centre, orange = general hospital, grey = regional referral hospital.

By level of care, NMS supplied the most quantity of antimicrobials to Level IV health centers, followed by general hospitals, regional referral hospitals in 2017, 2018, and 2019, respectively. National referral hospitals consumed the least quantity across the three study years, as shown in Figure 2. For JMS, district general hospitals consumed the most antimicrobials, followed by Level IV health centers and lastly regional referral hospitals, as seen in Figure 3. There was no record of JMS supplying antimicrobials to national referral hospitals. 

### 3.5. Antimicrobial Consumption by AWaRe Categorization

The quantities of antimicrobials supplied by NMS and JMS to study health facilities were analyzed by the WHO AWaRe categorization as presented in Table 3. Over the three years, on average, JMS distributed 65% in the access category, 35% in the watch category, and none for the reserve antimicrobials. On average, NMS distributed 81% access antimicrobials, 19% watch Antimicrobials, and 0.1% reserve antimicrobials.

When analyzed by level of care, for facilities supplied by NMS, national referral hospitals consumed 49% access, 47% watch, and 3% reserve antimicrobials. At regional referral level, 61% were consumed access, 37% watch, and 2% reserve antimicrobials. General hospitals consumed majorly access antimicrobials (79%), 20% watch, and only 0.1% reserve. Most antimicrobials at Level IV health center were access (80%), followed by watch at 20%, and no reserve antimicrobials.

### 3.6. Antimicrobial Consumption Density at Different Levels of Care Using Hospital Activity Units

The antimicrobial consumption density was calculated only for health facilities receiving medicines from NMS as the data were readily available in the National Health Monitoring Information System (HMIS). The denominators used were patient days and admissions per year. There is a gradual increase in average DDD per 100 patient days and average DDD per 100 admissions at each level of care from 2017 to 2019. Additionally, the antimicrobial density in each year of study increased from the higher level of care at national referral hospitals to lower levels of care with the health centers, level 4 recording the highest average DDD/100 patient days and highest average DDD/100 admissions as shown in Table 4. 

### 3.7. Analysis of Antimicrobial Consumption by Pharmacological Subgroup

When analyzed by pharmacological subgroup for consumption in inpatient care facilities, penicillin and the beta-lactam antibacterials were most used with 41%, 42%, and 36% consumption in 2017, 2018, and 2019, respectively. The other commonly consumed pharmacological groups from NMS were tetracyclines, quinolones, sulphonamides, and trimethoprim (Table 5). From JMS, the most consumed pharmacological classes were penicillin and beta-lactam antibacterials (J01C), macrolides (J01F), quinolones (J01M), and other beta-lactam antibacterials J01D (Table 5).

## 4. Discussion

This study is one of the first of its kind in Uganda that analyzes the total quantities of antimicrobials supplied by NMS and JMS to health facilities in DDDs and by AWaRE categorization and calculates AMC density as antimicrobial exposure per hospital inpatient activity units. There was an increase in total DDDs of antimicrobials supplied by NMS in 2019, whereas the total antimicrobial DDDs supplied by JMS did not change over the study years. Additionally, NMS supplied eight-fold as much antimicrobials as JMS, the reason for the difference lies in the higher annual turnover of NMS as compared to JMS because NMS receives more funding from government (USD 97.5 million in financial year 2019/2020) than JMS (USD 24.7 million) [17,18,19]. Among oral antibiotics, amoxicillin accounted for almost half of the antimicrobials used, whereas ceftriaxone accounted for over half of the parenteral antimicrobials. 

The majority of antimicrobials supplied to the public health facilities were from the access category of the WHO AWaRe classification, followed by watch antimicrobials, and a small proportion of reserve antimicrobials. Only higher-level facilities, at national referral and regional referral level received reserve antimicrobials from NMS. Level IV health centers consumed the most quantities of antimicrobials supplied by NMS, whereas hospitals consumed the most quantity of antimicrobials supplied by the JMS. Antimicrobial consumption density at health facilities showed a gradual increase from 2017 to 2019 and an even bigger increase as the level of care of the health facilities lowered. Analysis by pharmacological subclass showed that penicillins accounted for the largest proportion of DDD consumed. There was no significant difference in the mean DDD per 100 patient days both within the different levels of care and across the different years. 

The WHO report on surveillance of antimicrobial consumption 2016–2018 has reported antimicrobial exposure using DDD per 1000 population [21]. Hospital activity data provides more actual information on antimicrobial use within the facilities, allows for intra-facility or interfacility comparisons by comparing trends, and measures the impact of antimicrobial stewardship interventions.

Amoxicillin was the topmost distributed antimicrobial by both warehouses in all three years of the study. As amoxicillin is the first line recommended drug for the majority of simple infections in Uganda, such as bacterial pneumonia, dental infections, and ear infections, this could explain the high distribution. This use may also indicate overuse as other studies in Uganda have shown that antibiotics are prescribed for non-bacterial infections such as fevers attributed to malaria and upper respiratory tract infections of viral origin [22]. 

Similarly, ceftriaxone represented more than half of all injectable antimicrobials used in the three years (Table 2). This is aligned with reports showing ceftriaxone overuse in Ugandan hospitals whereby two thirds of patients in Uganda are prescribed ceftriaxone [23]. This indicates a need for stewardship interventions targeted at proper use of ceftriaxone, and providing alternatives, as ceftriaxone is regarded as a “Watch” antimicrobial by WHO [12]. A study in South Africa demonstrated that antibiotic stewardship programs reduced antimicrobial consumption over four years [24], and these practices could be emulated in Ugandan facilities. In the same way, macrolides and quinolones represented a substantial proportion of antimicrobial consumption, which raises concern as these are classified “Watch” category antimicrobials, and should, therefore, be used cautiously so that their effectiveness can be preserved. Interestingly, nitrofurantoin made the list of top 10 most used antibacterials in 2019. This likely reflects the change in national policy to use nitrofurantoin as first-line treatment of urinary tract infections, which underscores the impact guidelines can have on prescription practices and, therefore, procurement by suppliers. 

Analysis by pharmacological subclass showed that beta-lactam penicillins accounted for largest DDD consumed, which is a similar finding as reported in the four African countries of Burundi, Burkina Faso, Tanzania, and Cote d’Ivoire in the WHO 2018 report [21]. 

Level IV health centers consumed the most antimicrobials by volumes supplied by NMS, while hospitals consumed the most antibiotics supplied by the private-not-for-profit warehouses. This could be explained by the fact that while Level IV health centers provide secondary care, they are numerous compared to the higher-level facilities and, therefore, their pooled consumption may appear high. The antimicrobial consumption shows, when analyzing DDD/100 admissions and DDD/100 patients, a decreasing trend from HC IV, hospital, regional referral, and a small amount at national referral level. This demonstrates more antimicrobial exposure in terms of numbers at the lower levels, which could explain that the lower levels treat more community-acquired infections. In contrast, regional and national referral hospitals cater to more complicated illnesses that span beyond just infectious diseases to complex conditions such as non-communicable illnesses including heart diseases, diabetes, kidney diseases among others. 

With the AWaRe analysis, Access antibiotics accounted for two-thirds of the total DDDs consumed, which is in line with the WHO guidance, whereby Access antibiotics should constitute at least 60% of total consumption [25]. Lower-level public health facilities did not use any Reserve antimicrobials because there is restricted supply on which items they can purchase with government funds based on the national essential medicines list [26]. This finding could also be explained by budgetary limitations, meaning that health facilities cannot afford the Watch and Reserve antibiotics even though they need them. It has been shown before that LMIC still struggle with access to essential antibiotics [27]. The picture was slightly different for antimicrobials distributed by JMS, where the majority were of Access and Watch categories, and there were no Reserve antimicrobials reported. This could be due to the warehouse’s purchasing practices, and because the facilities supplied have the liberty to purchase from other privately-owned distributors. The results could have been different if data were collected from the health facilities themselves.

One of the reasons for conducting antimicrobial consumptions studies is to enable comparison with reports from national laboratory surveillance of AMR, and to draw relationships between sensitivity and resistance profiles of microorganisms and burden of antibiotics use. In Uganda, two studies conducted at tertiary hospitals and one in communities from urban and rural districts found high resistance of *Eschericia coli* to cotrimoxazole, amoxicillin and clavulanate (70–88%), and moderate resistance to fluoroquinolones and ceftriaxone (3–11%) [6,28,29]. Cotrimoxazole appears in the top 10 consumed antibiotics from this study, though it is mostly used as prophylaxis against opportunistic infections among HIV/AIDS positive populations and is no longer recommended as first line treatment for many diseases in Uganda. *Staphylococcus aureas* was also found to be multi-drug resistant to first line antibiotics [6] such as amoxicillin clavulanate and cotrimoxazole which are among the most consumed, and this narrows the therapeutic choices available to treat community-acquired infections. It is recognized that both laboratory surveillance systems and AMC studies are in their infancy in Uganda [30], but efforts to strengthen them are ongoing, and triangulation with AMC studies should yield more in-depth discussions in the future. This will, in turn, inform recommendations for national standard treatment guidelines so that prescriptions are based on antibiogram guidance at the health facility level, and from the supply chain side, this will enable alignment of procurement and distribution of the most appropriate antibiotics to the different levels of care. 

This study has several implications on policy and practice of AMR consumption in Uganda. This study represents a baseline information on the antimicrobial consumption in health facilities in Uganda for three years, and will be useful for future studies to monitor annual trends in consumption. This will help to evaluate the effectiveness of stewardship programs within the health facilities. As Uganda does not have an established system for AMC surveillance yet, our study methodology can be used in national efforts to create AMC surveillance systems and to study AMC at national level and within the private-for-profit sector. 

There were some limitations to this study. First, the study only assessed national warehouses; the private-for-profit sector distributors’ sales data were not analyzed, as this information is not readily available due to the distributors’ hesitancy and concerns about revealing their sales data. This may have caused the antimicrobial consumption to be underestimated in this report. However, analysis of antimicrobial imports and domestically manufactured products would provide a more complete picture of antimicrobial exposure of the Ugandan population at national level as it incorporates the contribution of private sector. Second, the study did not consider facilities that provide primary care on an outpatient basis and community consumption as the methodology to measure density only provided for facilities with inpatient care. Third, the methodology also provided aggregate consumption data as a proxy for use but does not necessarily reflect the health facilities’ prescribing practices. Lastly, there were some antibiotic combinations that did not have ATC codes assigned to them, such as flucloxacillin and amoxicillin combination, and therefore were left out of the analysis, which could have led to underestimation of the total DDDs consumed. 

## 5. Conclusions

Our study showed that there could be a strong link between antimicrobial consumption and standard treatment guidelines, which can be explored as a venue to promote appropriate antimicrobials use. The study also revealed high use of ceftriaxone, which needs to be protected as a Watch antimicrobial through proactive stewardship activities. This study also provided baseline data for future studies on trends in the pattern of antimicrobial use in Uganda. Finally, the study methodology can be used to compare consumption across health facilities within the same and varying levels of care and in contexts similar to Uganda, and help to set up a standardized system for continuous monitoring of antibiotic consumption, and also contribute to the global WHO/GLASS surveillance system for AMC data.

## Figures and Tables

**Table 2:** Top 10 antimicrobials in relation to overall defined daily doses (DDD) distributed by National Medical Stores and Joint Medical Stores of Uganda from 2017 to 2019.

	2017		2018		2019	
Ranking	Antibiotic	% of DDD	Antibiotic	% of DDD	Antibiotic	% of DDD
***National Medical Stores***						
**1**	Amoxicillin	38.7%	Amoxicillin	38.3%	Amoxicillin	26.8%
**2**	Metronidazole	15.6%	Metronidazole	17.4%	Cotrimoxazole	19.9%
**3**	Doxycycline	14.7%	Doxycycline	12.1%	Metronidazole	12.2%
**4**	Ciprofloxacin	10.4%	Ciprofloxacin	10.1%	Doxycycline	9.8%
**5**	Cotrimoxazole	9.0%	Cotrimoxazole	6.3%	Ampicillin-Cloxacillin	8.6%
**6**	Ceftriaxone	2.8%	Ceftriaxone	4.2%	Ciprofloxacin	7.2%
**7**	Erythromycin	2.2%	Erythromycin	2.9%	Azithromycin	5.3%
**8**	Gentamicin	1.5%	Ampicillin - Cloxacillin	2.3%	Erythromycin	2.2%
**9**	Ampicillin-Cloxacillin	1.4%	Gentamicin	1.4%	Ceftriaxone	2.1%
**10**	Levofloxacin	1.1%	Levofloxacin	1.2%	Nitrofurantoin	1.8%
***Joint Medical Stores***						
**1**	Amoxicillin	15.8%	Amoxicillin	18.1%	Amoxicillin	18.5%
**2**	Ciprofloxacin	12.4%	Doxycycline	11.6%	Doxycycline	9.8%
**3**	Doxycycline	10.2%	Metronidazole	10.4%	Metronidazole	9.7%
**4**	Metronidazole	9.8%	Ciprofloxacin	10.2%	Ampicillin-Cloxacillin	9.6%
**5**	Ampicillin-Cloxacillin	8.2%	Ampicillin-Cloxacillin	7.4%	Ciprofloxacin	9.1%
**6**	Azithromycin	7.8%	Erythromycin	6.5%	Azithromycin	7.2%
**7**	Erythromycin	6.9%	Ceftriaxone	6.3%	Ceftriaxone	7.0%
**8**	Ceftriaxone	5.8%	Azithromycin	6.3%	Erythromycin	5.5%
**9**	Gentamycin	3.1%	Cloxacillin	3.5%	Cloxacillin	3.3%
**10**	Cefixime	3.0%	Gentamycin	3.0%	Nitrofurantoin	3.3%

**Table 3:** Proportion of antibiotics consumed by AWaRe category by National Medical Stores and Joint Medical stores in Uganda, 2017–2019.

AWaRe	Total DDD					
	2017 sum	%	2018 sum	%	2019 sum	%
	*National Medical Stores*					
**Access**	24,282,698.7	82.0	23,620,557.4	80.1	26,980,972.2	80.2
**Watch**	5,317,560.6	17.9	5,842,719.4	19.8	6,651,496.5	19.8
**Reserve**	450.0	0.001	6095.0	0.0	7300.0	0.02
**Other**	3629.0	0.012	3825.0	0.01		
	***Joint Medical Stores***					
**Access**	2,495,735.4	61.8	2,603,915.4	65.6	2,681,477.7	65.9
**Watch**	1,543,095.2	38.2	1,363,196.3	34.4	1,387,103.6	34.1

**Table 4:** Average antimicrobial defined daily doses (DDD) per 100 patient days and per 100 admissions supplied by NMS in Uganda, stratified by consumption at the level of care, 2017–2019.

Level of Care	Average DDD/100 Patient Days			Average DDD/100 Admissions		
	2017	2018	2019	2017	2018	2019
National Referral Hospital	99.9	113.3	191.1	1259.2	1417.9	2079.9
Regional Referral Hospital	570.6	559.3	523.9	2302.3	2435.4	2004.9
General Hospital	978.2	1124.4	1080.9	3090.9	3340.1	3101.7
Health Centre Level IV	3536.9	2852.5	4044.5	4382.6	4305.8	3776.7

**Table 5:** Proportion of antimicrobials consumed by pharmacological subgroup (ATC *) per calendar year in public health facilities with inpatient care stratified by distribution from National Medical Stores and Joint Medical Stores in Uganda during 2017–2019.

ATC *	Level: Pharmacological Class	Percentage of DDD		
		2017	2018	2019
***National Medical Stores***				
J01A	Tetracyclines	14.7	12.1	9.8
J01B	Amphenicols	0.3	0.2	0.1
J01C	Beta-Lactam antibacterials, Penicillins	40.8	41.6	36.2
J01D	Other beta-lactam antibacterials	3.7	4.8	4.0
J01E	Sulfonamides and trimethoprim	9.0	6.3	19.9
J01F	Macrolides	2.6	3.2	7.8
J01G	Aminoglycosides	1.6	1.4	0.1
J01M	Quinolone antibacterials	11.5	11.7	7.9
J01X	Other antibacterials	15.8	18.6	14.1
***Joint Medical Store***				
J01A	Tetracyclines	10.2	11.6	9.8
J01B	Amphenicols	0.3	0.3	0.4
J01C	Beta-Lactam antibacterials, Penicillins	33.1	34.4	37.1
J01D	Other beta-lactam antibacterials	11.6	11.5	11.4
J01E	Sulfonamides and trimethoprim	1.5	1.6	1.7
J01F	Macrolides	14.8	12.8	12.7
J01G	Aminoglycosides	3.1	3.0	2.3
J01M	Quinolone antibacterials	13.1	11.4	11.0
J01X	Other antibacterials	11.9	18.6	13.1
P01A	Antiprotozoals against amoebiasis	0.3	0.3	0.3
J01A	Tetracyclines	10.2	11.6	9.8
J01B	Amphenicols	0.3	0.3	0.4

* ATC—Anatomical Therapeutic Chemical classification.

## Data Availability

Data used for the analysis in the study are available with the corresponding authors upon request, and on provision of reasonable justification for its further use.

## Appendix A

**Table A1:** Antimicrobial products supplied by National Medical Stores (NMS) and Joint Medical Stores (JMS) from 2017 to 2019.

	Product Name	Pack Size	Paediatric	** AWaRe	Warehouse	Total DDD 2017–2019
	**Nitroimidazole derivatives (P01AB)**					
1	Metronidazole tablet 200 mg tablet	100, 1000	No	Access	Both	14,055,650
2	Metronidazole oral suspension 200 mg/5 mL	100 mL bottle	Yes	Access	Both	95,602
3	Metronidazole rectal suppositories 500 mg	10 supp	No	Access	NMS	4950
4	Metronidazole infusion, 500 mg/100 mL	100 mL bottle	No	Access	Both	938,357
5	Ornidazole injection, bottle 500 mg/100 mL	100 mL bottle	No	Access	Both	7475
6	Tinidazole tablet 500 mg	100	No	Access	NMS	28,725
7	Secnidazole tablet 1 G	2	No	Access	JMS	7806
	**Tetracyclines (J01A)**					
8	Doxycycline capsules 100 mg	100	No	Access	Both	12,533,400
	**Amphenicols (J01B)**					
9	Chloramphenicol capsule 250 mg capsule	100, 1000	No	Access	Both	124,920
10	Chloramphenicol oral suspension 125 mg/5 mL	100 mL bottle	Yes	Access	JMS	3004
11	Chloramphenicol injection, vial 1 G	50 vials	No	Access	Both	93,706
	**Penicillins, betalactams (J01C)**					
12	Amoxicillin capsule 250 mg	1000, 100	No	Access	Both	31,689,473
13	Amoxicillin dispersable tablets 125 mg	20, 100	Yes	Access	Both	50,427
14	Amoxicillin dispersable tablets 250 mg	10, 20, 100	Yes	Access	NMS	2,110,359
15	Amoxicillin powder for suspension 125 mg/5 mL	100 mL bottle	Yes	Access	JMS	78,260
16	Amoxicillin clavulanate injection 1.2 G	1 vial	No	Access	Both	1356
17	Amoxicillin clavulanate tablet 1000 mg	10, 14	No	Access	JMS	4672
18	Amoxicillin clavulanate syrup 156 mg/5 mL	100 mL bottle	Yes	Access	Both	937
19	Amoxicillin clavulanate tablet 228.5 mg tablet	10	Yes	Access	JMS	552
20	Amoxicillin clavulanate syrup 228.5 mg/5 mL	100 mL bottle	Yes	Access	JMS	57,798
21	Amoxicillin clavulanate tablet 625 mg tablet	14, 15, 16, 20	No	Access	Both	66,615
22	Amoxicillin/clavulanic acid tablet 375 mg	10, 20, 100	No	Access	JMS	43,710
23	Ampicillin injection, vial 500 mg	100 mL bottle	No	Access	Both	290,074
24	Ampicillin oral suspension 125 mg/5 mL	100 mL bottle	Yes	Access	JMS	3021
25	Ampicillin capsules 250 mg	100	No	Access	JMS	613
26	Ampicillin/cloxacillin injection, vial 250 mg/250 mg	1, 100 vials	No	Access	NMS	2,871,400
27	Ampicillin/cloxacillin capsules 250 mg/250 mg	100, 200	No	Access	NMS	4,438,100
28	Ampicillin/cloxacillin oral suspension 125 mg/5 mL	100 mL bottle	Yes	Access	JMS	121,550
29	Benzathine benzylpenicillin injection, vial 2.4 mu/1.44 g	10, 50 vials	No	Access	Both	89,388
30	Benzylpenicillin injection, vial 1 MU/600 mg	10, 50 vials	No	Access	Both	483,027
31	Cloxacillin capsules 250 mg	100	No	Access	Both	338,363
32	Cloxacillin injection, vial 500 mg	50 vials	No	Access	Both	127,688
33	Phenoxymethylpenicillin tablets 250 mg	100	No	Access	JMS	84,763
34	Piperacillin-tazobactam injection, vial 4.5 g	1 vial	No	Watch	Both	63,730
35	Procaine benzylpenicillin injection, vial 4 MU	10, 50	No	Access	NMS	24,160
36	Flucloxacillin/amoxicillin oral suspension 250 mg	80 mL bottle	No	Access	Both	*
37	Flucloxacillin/amoxicillin capsules 250/250 mg	16	No	Access	Both	*
38	Flucloxacillin/amoxicillin injection, vial 500 mg/500 mg	1 vial	No	Access	Both	*
	**Carbapenems (J01DH)**					
39	Imipenem/cilastatin injection, vial 500/500 mg	1 vial	No	Watch	NMS	3
40	Meropenem injection, vial 500 mg	1 vial	No	Watch	NMS	21,391
	**Cephalosporins (J01D)**					
41	Cefixime tablets 200 mg	10, 12, 20, 120	No	Watch	Both	491,015
42	Cefixime capsules 400 mg	5, 6, 100	No	Watch	Both	44,489
43	Cefixime oral suspension 50 mg/5 mL	100 mL bottle	Yes	Watch	JMS	37,513
44	Cefixime oral suspension 100 mg/5 mL	50, 60 mL bottle	Yes	Watch	JMS	7112
45	Cefotaxime injection, vial 1 G	1 vial	No	Watch	Both	9563
46	Cefotaxime injection, vial 500 mg	1 vial	No	Watch	NMS	34,718
47	Cefpodoxime tablets 200 mg	10	No	Watch	JMS	3140
48	Cefpodoxime suspension 50 mg/5 mL	100 mL bottle	Yes	Watch	JMS	455
49	Ceftriaxone injection, vial 1 G	1 vial	No	Watch	Both	3,536,638
50	Ceftriaxone injection, vial 500 mg	1 vial	No	Watch	JMS	1303
51	Ceftriaxone + sulbactam injection, vial 1.5 G	1 vial	No	Watch	JMS	5297
52	Cefuroxime tablets 250 mg	10	No	Watch	JMS	9660
53	Cefuroxime tablets 500 mg	10, 100	No	Watch	Both	830,940
54	Cefuroxime injection, vial 1.5 G	1 vial	No	Watch	NMS	650
55	Cefuroxime injection, vial 750 mg	1 vial	No	Watch	NMS	250
56	Cefuroxime suspension 125 mg/5 mL	50, 70, 100 mL bottle	Yes	Watch	Both	44,180
57	Cephalexin capsules 250 mg	100	No	Access	JMS	156,250
58	Cephalexin oral suspension 125 mg/5 mL	100 mL bottle	Yes	Access	JMS	21,318
	**Sulphonamides and** ** Trimethoprim (J01E)**					
59	Cotrimoxazole tablet 120 mg	100, 1000	Yes	Access	Both	35,904
60	Cotrimoxazole tablet 480 mg	100, 1000	No	Access	Both	4,902,800
61	Cotrimoxazole tablet 960 mg	100, 1000	No	Access	Both	6,474,350
62	Cotrimoxazole oral suspension 240 mg/5 mL	60, 100 mL bottle	Yes	Access	JMS	14,593
63	Cotrimoxazole injection, vial 80 mg/400 mg	1 vial	No	Access	NMS	100
	**Macrolides and Lincosamides (J01F)**					
64	Azithromycin tablet 250 mg	180, 500	No	Watch	NMS	791,218
65	Azithromycin tablet 500 mg	3, 6, 30, 90	No	Watch	Both	2,009,970
66	Azithromycin oral suspension 200 mg/5 mL	15 mL bottle	Yes	Watch	JMS	30,712
67	Erythromycin oral suspension 125 mg/5 mL	100 mL bottle	Yes	Watch	Both	102,613
68	Erythromycin tablet 250 mg	100, 1000	No	Watch	Both	2,895,650
69	Clarithromycin tablet 500 mg	7, 14	No	Watch	Both	150,451
70	Clindamycin capsules 150 mg	50	No	Access	JMS	4794
	**Aminoglycosides (J01G)**					
71	Amikacin Injection vial 500 mg/2 mL	1 vial	No	Access	NMS	16,612
72	Gentamicin injection, ampoule 80 mg/2 mL	100 vials	No	Access	Both	1,171,049
73	Kanamycin injection, ampoule 1 G	10, 50 vials	No	Watch	NMS	19,740
74	Streptomycin injection, vial 1 G	50, 100 vials	No	Watch	NMS	69,600
	**Fluoroquinolones (J01M)**					
75	Ciprofloxacin tablet 500 mg	10, 100	No	Watch	Both	9,671,035
76	Ciprofloxacin tablet 750 mg	20	No	Watch	JMS	10,125
77	Ciprofloxacin tablet 250 mg	100	No	Watch	JMS	11,075
78	Ciprofloxacin injection, 200 mg/100 mL	100 mL bottle	No	Watch	Both	49,688
79	Levofloxacin tablet 250 mg	100	No	Watch	NMS	841,500
80	Levofloxacin tablet 500 mg	5, 10 tablets	No	Watch	Both	136,010
81	Levofloxacin tablet 750 mg	10	No	Watch	JMS	11,775
82	Levofloxacin injection, bottle 500 mg/150 mL	150 mL bottle	No	Watch	Both	12,221
83	Levofloxacin injection, bottle 750 mg/100 mL	100 mL bottle	No	Watch	NMS	1860
84	Moxifloxacin tablet 400 mg	100	No	Watch	NMS	181,600
85	Nalidixic acid tablet 500 mg	100, 1000	No	Watch	Both	19,975
86	Ofloxacin injection, bottle 200 mg/100 mL	200 mL bottle	No	Watch	NMS	4730
87	Ofloxacin tablet 200 mg	100	No	Watch	JMS	432
88	Pefloxacin tablet 400 mg	100	No	Watch	JMS	3950
89	Ofloxacin and ornidazole tablets 200/500 mg	100	No	Watch	JMS	13,110
	**Glycopeptides, Nitrofuran, Linezolid (J01X)**					
90	Linezolid tablet 600 mg	10, 20, 100	No	Reserve	NMS	13,845
91	Nitrofurantoin tablet 100 mg	100, 1000	No	Access	Both	1,352,050
92	Vancomycin injection, vial 500 mg	1 vial	No	Watch	NMS	128

* DDD not calculated as Flucloxacillin/Amoxicillin has no ATC/DDD assigned. ** AWaRe—access, watch, reserve.
